# *Blastocystis*, an Enigmatic Parasite

**DOI:** 10.1007/s11686-026-01331-z

**Published:** 2026-07-13

**Authors:** José Cebrián-Carmona, José Antonio Garrido-Cárdenas, Concepción Mesa-Valle

**Affiliations:** Department of Biology and Geology, University of Almería, Almería, 04120 Spain

**Keywords:** *Blastocystis* spp., Bibliometric analysis, Molecular epidemiology, Subtypes (STs), PCR and sequencing, Microbiota.

## Abstract

*Blastocystis* spp. is the most prevalent human enteric protist, yet its clinical significance remains controversial. Currently recognized as a genetically diverse stramenopile with zoonotic potential, it comprises at least 42 subtypes, of which ST1–ST4 account for most human infections. Despite more than a century of research, its pathogenic role and ecological interactions are still debated. We performed a comprehensive bibliometric analysis of global scientific production on Blastocystis spp. from 1917 to 2025 using the Scopus database, retrieving 3710 publications. Temporal trends, geographic distribution, leading authors and institutions, and thematic evolution were assessed through keyword normalization and classification into five categories: classical diagnosis, PCR/molecular approaches, genetics/phylogeny, clinical/epidemiology, and microbiota. Results indicate a steady increase and a subsequent acceleration in publication trends since the late 1980s, with a compound annual growth rate of 11.56% between 1988 and 2025. Early research was dominated by microscopy-based diagnosis, whereas the introduction of PCR and sequencing technologies reshaped the field by enabling subtype identification and phylogenetic analysis. Since 2010, clinical and epidemiological studies have expanded significantly, and microbiota-oriented research has emerged as a growing line of investigation. Co-occurrence analysis indicates that Blastocystis spp. is frequently studied in the context of polyparasitism and intestinal coinfections. Overall, Blastocystis spp. research has evolved from descriptive parasitology to a multidisciplinary field integrating molecular, clinical and ecological approaches, highlighting the need for context-dependent models of host–parasite–microbiota interactions.

## Introduction

*Blastocystis* spp. is the most common enteric parasite in the human population [1]. First observed in 1849 by Brittan and Swayne as “cholera bodies,” the parasite was later independently named *Blastocystis* by Alexeieff in 1911 and *hominis* by Brumpt in 1912, who initially classified it as a harmless saprophytic yeastAlexeieff [2], Brumpt [3].

Due to the presence of phenotypic traits intermediate between distinct groups of organisms, its taxonomic classification has represented a major challenge for decades. Currently, *Blastocystis* spp. is recognized as a zoonotic parasite with low host specificity and extensive genetic diversity structured into distinct subtypes (STs) [4]. The organism was recognized as a protist by Zierdt CH et al. [5] and later placed within the eukaryotic phylum Heterokontophyta (kingdom Chromista) by Silberman et al. in 1996 based on SSU rRNA gene analysis Silberman et al. [6].

Numerous molecular studies have since confirmed that *Blastocystis* spp. is a heterokont (also known as a stramenopile or “botanical protist”). Unlike most members of this group, however, *Blastocystis* spp. lacks flagella (or at least none have been observed in any life stage), and its mitochondria possess tubular cristae, despite its anaerobic metabolism, whose functional pathways remain incompletely understood [6].

To date, 42 subtypes have been described in various hosts, with ST1–ST4 accounting for approximately 90% of human infections worldwide [7–9]. Nevertheless, prevalence patterns are influenced by factors such as geographic location, host age and immune status [10].

Since the late twentieth century, an increase in *Blastocystis* spp. infections associated with diarrhoea has been reported among travellers, children, animal handlers, and immunocompromised populations [11]. Given the growth of immunosuppressive disorders, establishing this relationship has become increasingly important. Several studies indicate that *Blastocystis* spp. can cause opportunistic infections in patients with cancer [12, 13], transplant recipients [14, 15] and individuals with AIDS [16, 17], where impaired T-cell responses likely favour higher prevalence rates.

Over the last decades, approximately 60% of published studies on *Blastocystis* spp. have focused on its potential pathogenic role [1]. Clinical manifestations include mild and self-limiting, acute, or chronic diarrhoea, alongside abdominal pain, flatulence, nausea, vomiting, and non-specific gastrointestinal discomfort [18]. In vitro and animal models demonstrate that while the parasite does not invade the intestinal mucosa, it induces alterations in intestinal permeability through the secretion of cysteine proteases, degrades secretory IgA, and triggers host-cell apoptosis [19, 20]. Consequently, *Blastocystis* spp. has been linked to gastrointestinal disorders such as irritable bowel syndrome (IBS) and ulcerative colitis [21].

The enigmatic nature of this parasite has also stimulated growing interest in its potential association with human colorectal cancer, where systematic meta-analyses report its prevalence among cancer patients, suggesting cytopathic and immunomodulatory effects that might aggravate colorectal carcinogenesis [21, 22]. Additionally, an increasing number of studies have linked *Blastocystis* spp. infection to allergic diseases like asthma and chronic urticaria, potentially triggered by elevated IgE responses against intestinal allergens [23, 24].

Conversely, other investigations report higher *Blastocystis* spp. prevalence in healthy populations compared with individuals suffering from ulcerative colitis or irritable bowel syndrome [20,25]. However, its detection in patients with irritable bowel syndrome, often in co-infection with other pathogens, continues to fuel discussions regarding its clinical relevance [26]. This discrepancy is increasingly studied in the context of the human gut microbiota. Accumulating evidence suggests that *Blastocystis* spp. colonization is generally associated with a healthy gut microbiota and higher bacterial diversity [27, 28]. However, this interaction is bidirectional, as the surrounding microbiota may also modulate parasite traits and promote pathogenic behavior, fueling the debate over whether it functions as a pathogen or a commensal organism, highlighting the need to further elucidate the nature of this association [29–31]. This is particularly relevant in vulnerable populations such as children, where infection has been profile in cases of acute diarrhea or linked as a potential risk factor for micronutrient malabsorption and iron-deficiency anemia [32, 33].

Despite the growing volume of research on *Blastocystis* spp., significant gaps remain regarding its precise epidemiological patterns and subtype distribution in specific regions, which hinders a comprehensive understanding of its clinical impact and transmission dynamics [34, 35]. The present study aims to characterize global research trends and thematic evolution in *Blastocystis spp*. literature through a bibliometric analysis.

## Materials and Methods

### Data Sources and Search Strategy

The development of the digital era has led to the creation of numerous online scientific databases that enable specialized searches and bibliometric analyses on specific topics. Among the most widely used platforms in the biomedical field are Scopus, Web of Science, PubMed and Google Scholar. PubMed is primarily focused on medicine and biomedical sciences, whereas Scopus and Web of Science cover a broader range of disciplines and allow the export of metadata for systematic analysis.

For the present study, the Scopus database (Elsevier) was selected, as it is currently the largest abstract and citation database of peer-reviewed scientific literature. As of 2025, Scopus indexes more than 43,000 titles from scientific journals, books and conference proceedings, originating from approximately 12,000 international publishers. The platform allows the application of multiple filters (author, affiliation, country, year, document type and keywords), making it particularly suitable for large-scale bibliometric studies.

The bibliographic search was conducted in the Scopus database using the following query:

TITLE-ABS-KEY (*Blastocystis* OR blastocystosis). This search strategy retrieves documents in which the terms appear in the title, abstract or keywords. No restrictions were applied regarding language or year of publication, allowing coverage from the earliest indexed records (1917) through 2025. As a result, a total of 3710 publications related to *Blastocystis* and blastocystosis were initially retrieved.

It should be noted that bibliometric results are influenced by the design of the search strategy, as well as by potential updates or changes in the database indexing algorithms. Furthermore, keywords assigned by authors or publishers may not always accurately reflect the full content of an article, which represents a common methodological limitation in bibliometric analyses. In addition, bibliometric analyses rely on the accuracy and consistency of indexed metadata; consequently, variations in keyword assignment by authors or publishers, as well as changes in indexing practices over time, may affect the representation of specific research topics. These limitations are common to bibliometric studies and should be considered when interpreting the results.

Furthermore, while Scopus provides the most comprehensive coverage of peer-reviewed metadata for large-scale bibliometric mapping, the exclusion of other databases such as PubMed, Embase, or Web of Science, as well as grey literature and regional databases, constitutes a recognized methodological limitation. Consequently, some regional or non-indexed publications may be underrepresented, introducing a potential retrieval bias that should be considered when interpreting the global trajectories of the field.

### Keyword Analysis

For the keyword analysis, synonymous terms and lexical variants (e.g., *Blastocystis hominis* and *B. hominis*) were grouped, and generic, non-informative terms (such as “study”) were removed. Several aspects were examined, including the temporal evolution of the number of publications per year, the geographic and institutional distribution of scientific output, collaboration networks and thematic trends.

### Keyword Processing and Cleaning

Keywords retrieved from Scopus records were initially processed and normalized using Microsoft Excel and its Power Query tool, enabling systematic data cleaning, transformation, and aggregation. When multiple keywords appeared grouped within the same field, they were separated to ensure that each term was analyzed independently. Basic normalization steps included removing unnecessary whitespaces and unifying obvious lexical variants. To reduce background noise and focus on consolidated thematic trends, keywords with a total frequency of fewer than 50 occurrences across the entire dataset were excluded. This threshold was applied following commonly accepted criteria in bibliometric studies to balance the inclusion of representative thematic trends while minimizing the influence of sporadic terms, thereby improving the interpretability of temporal trends.

### Thematic Classification of Keywords

The finalized keywords were grouped into five thematic categories based on their methodological or conceptual focus following a rule-based approach, in which keywords sharing similar analytical or biological meanings were assigned to the same category. The five categories were defined as follows:

- Classical diagnosis: including terms related to microscopy, staining techniques, and conventional parasitological methods.
- PCR/molecular: comprising keywords associated with molecular amplification and analysis techniques, such as PCR, DNA extraction, and basic sequencing approaches.
- Genetics/phylogeny: encompassing terms related to genetic variability, genotyping, subtype identification, and phylogenetic analyses.
- Clinical/epidemiology: including studies addressing prevalence, risk factors, symptomatology, coinfections, and clinical or population-based analyses.
- Microbiota: grouping keywords related to the intestinal microbiota, microbiome research, and metagenomic approaches.

This structured categorization allowed individual keywords to be integrated into broader conceptual blocks, facilitating a robust and interpretable analysis of the temporal evolution of research approaches in *Blastocystis* spp. studies.

### Temporal Analysis and Figure Construction

For each thematic category, the annual research output was calculated by aggregating the frequencies of the corresponding keywords across each calendar year, spanning from the first indexed publication through 2025. Data aggregation and trend analysis were executed using structured data matrices and plotted to visualize chronological shifts.

To trace the global evolution of the discipline, Fig. 5 presents a comprehensive overview that integrates all five thematic categories, illustrating how classical, clinical, and emerging methodologies have sequentially shaped the historical trajectory of *Blastocystis* spp. research. Conversely, to isolate and scrutinize the recent technological shift within the discipline, Fig. 6 focuses exclusively on a combined dataset of the PCR/molecular, genetics/phylogeny, and microbiota categories, providing a specialized assessment of the precise evolution and scaling of molecular-based approaches.

Graphical representations were standardized using line charts, with the X-axis representing the timeline of publication and the Y-axis indicating the quantitative volume of research per category.

### Methodological Considerations

The adopted approach enables the identification of robust trends in the evolution of *Blastocystis* spp. research while minimizing the influence of low-frequency terms and highlighting progressive shifts in diagnostic, molecular, clinical and ecological research approaches. This procedure is consistent with previously described bibliometric methodologies and is well suited for the longitudinal analysis of expanding research fields.

## Results

### Temporal Evolution of Scientific Production on *Blastocystis* spp

**Fig. 1:**
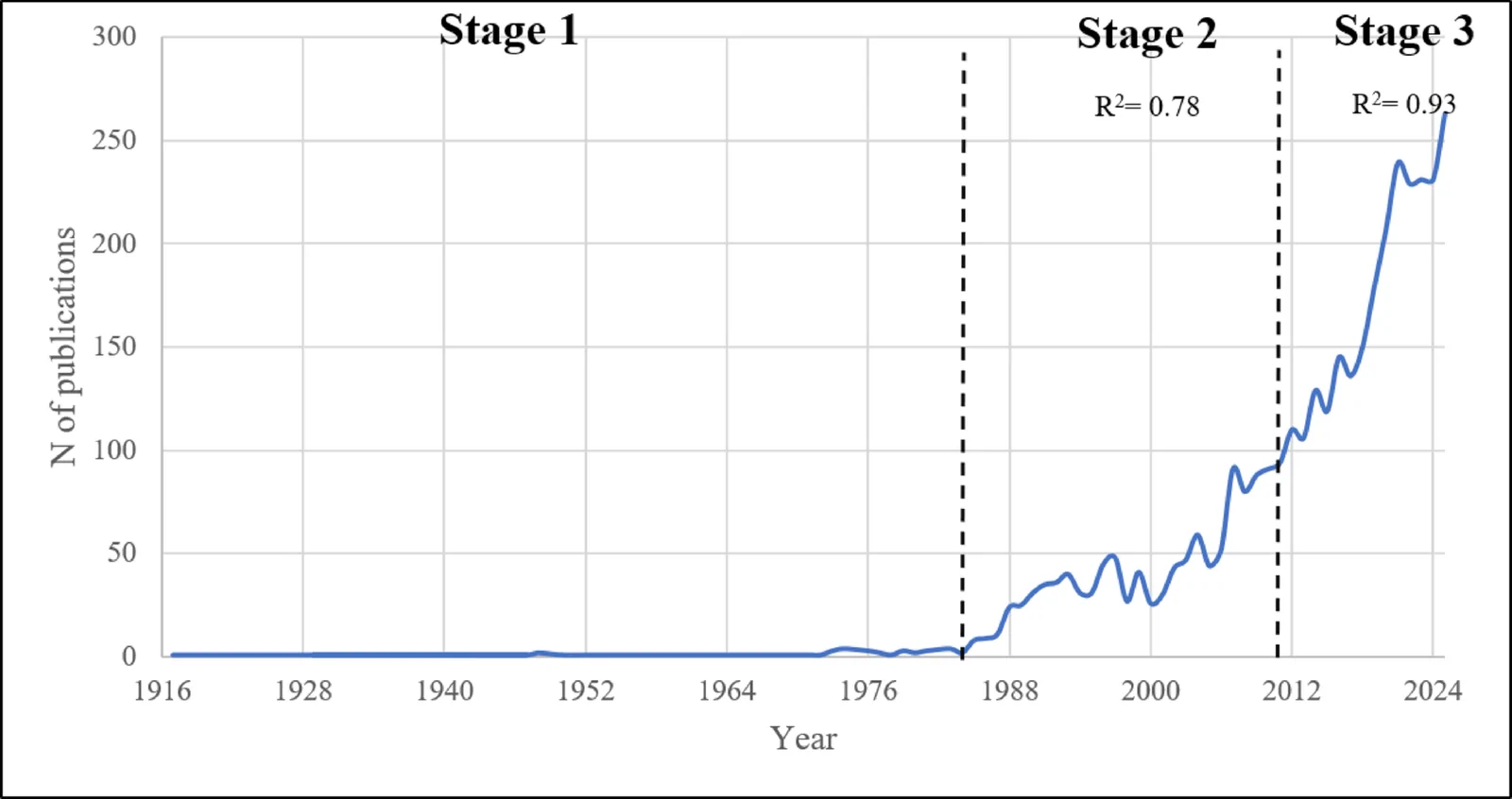
Annual evolution of the number of scientific publications on *Blastocystis* spp. between 1917 and 2025. Three temporal stages are indicated. Stage 2 shows a linear increase in publication output, while Stage 3 is characterized by a quadratic growth pattern, reflecting an accelerated expansion of research activity. The coefficient of determination (R²) indicates the goodness of fit of the regression models applied to each stage

After applying the established search and selection criteria, a total of 3,710 articles were identified. Figure 1 shows a clear upward trend in the number of scientific publications related to *Blastocystis* spp. over the last century. From its first mention in the scientific literature, publications were sporadic and scarce until the late 1980s. From that point onwards, a progressive increase can be observed, which becomes particularly pronounced from the year 2000, with a marked acceleration after 2010.

To better characterize the dynamics of publication growth, regression analyses were applied to the different temporal stages defined in Fig. 1. During Stage 2 (approximately 1988–2010), the increase in scientific output follows a linear trend, indicating a steady and sustained growth in the number of publications. This behavior is consistent with a first-degree polynomial model of the form:

* y=ax+b*

where *y* represents the number of publications and *x* the year. The corresponding coefficient of determination (R² ≈ 0.78) indicates that nearly 78% of the variability in publication output during this period is explained by the linear model, reflecting a relatively stable expansion of research activity.

In contrast, Stage 3 (from around 2010 onwards) is better described by a second-degree polynomial model, reflecting an acceleration in publication growth. This trend was fitted using a quadratic equation of the form:

$$\:y=a{x}^{2}+bx+c$$

The high coefficient of determination obtained for this stage (R² ≈ 0.93) indicates an excellent fit of the model to the observed data, with approximately 93% of the variance explained. This suggests a non-linear expansion of research output, characteristic of rapidly growing or consolidating research fields.

The most significant growth occurred during the last decade, especially between 2015 and 2022, when the annual number of publications exceeded 150. This expansion can be attributed to several factors, including advances in molecular diagnostic techniques, the growing interest in the intestinal microbiome, and the recognition of *Blastocystis* spp. as a frequent— and potentially pathogenic—component of the human gastrointestinal tract.

To quantitatively estimate the growth dynamics of the specialized literature on *Blastocystis* spp., the Compound Annual Growth Rate (CAGR) was calculated for the period between 1988 and 2025. The starting year was deliberately selected, as it coincides with the onset of molecular biology–based approaches in *Blastocystis* spp. research, particularly following the introduction and progressive adoption of polymerase chain reaction (PCR) techniques in parasitology and microbiology. Prior to this period, scientific output on *Blastocystis* spp. was sparse, irregular and largely based on classical diagnostic methods, which limits the interpretability of growth metrics.

The CAGR reflects the average annual expansion rate of publications over the selected interval, independently of year-to-year fluctuations. The resulting value of 11.56% indicates a markedly accelerated increase in research output, highlighting the consolidation of *Blastocystis* spp. as a relevant topic within global scientific production. Moreover, this metric facilitates comparison with other emerging biomedical research areas and further supports the need to continue investigating the clinical, molecular and ecological implications of *Blastocystis* spp., given the sustained and structured growth observed since the late 1980s.

### Authors, Institutions and Countries with the Highest Scientific Output on *Blastocystis* spp

Table 1 presents the ten most productive authors in the field of *Blastocystis* spp. research, each with at least 30 publications indexed in Scopus.

**Table 1:** The ten most productive authors in *Blastocystis* spp. research, indicating the number of indexed publications, main institutional affiliation and country

Author name	Nº of Publications	Affiliation	Country
Stensvold, C.R.	87	Statens Serum Institut	Denmark
Tan, K.S.W.	54	National University of Singapore	Singapore
Yoshikawa, H.	48	Nara Women´s University	Japan
Viscogliosi, E.	46	University of Lille/CNRS	France
Carmena, D.	44	National Center for Microbiology (ISCIII)	Spain
Singh, M.	40	Centers for Disease Control and Prevention (CDC)	United States
Köster, P.C.	40	University of Lille/CNRS	France
Yap, E.H.	34	University of Lille/CNRS	France
Dashti, A.	32	Centers for Disease Control and Prevention (CDC)	United States
Mirjalali, H.	31	Centers for Disease Control and Prevention (CDC)	United States

Nº, Number; CNRS, Centre National de la Recherche Scientifique; ISCIII, Instituto de Salud Carlos III; CDC, Centers for Disease Control and Prevention.

Among them, Christen Rune Stensvold, affiliated with the Statens Serum Institut in Copenhagen (Denmark), stands out for his extensive work on molecular subtyping and the global distribution of *Blastocystis spp*. Similarly, Kevin S. W. Tan, from the National University of Singapore, has made major contributions to the functional biology of the parasite and its potential immunological interactions with the host.

The individual contribution percentage (defined as the proportion of publications contributed by each author relative to the total number of publications analysed) varies across authors, with the highest values observed for Stensvold, C. R. (2.34%, Statens Serum Institut, Denmark) and Tan, K. S. W. (1.45%, National University of Singapore).

The authors listed in Table 1 are affiliated with institutions located in countries such as Denmark, Singapore, Japan, France, Spain and the United States. Although countries such as Iran, China and Turkey rank among the most productive at the institutional and national levels, their most prominent individual researchers do not appear among the top ten authors in terms of publication volume.

This distribution highlights not only the leadership of specific centres of excellence, but also the consolidation of international collaborative networks in *Blastocystis* spp. research. Particularly strong collaborations are observed among European institutions such as the Statens Serum Institut (Denmark), the University of Lille/CNRS (France) and the National Center for Microbiology (ISCIII, Spain), as well as links with the Centers for Disease Control and Prevention (United States) and Asian universities such as the National University of Singapore and Nara Women’s University. These collaborations have been instrumental in advancing molecular subtyping, global epidemiology and the understanding of host–parasite interactions.

**Fig. 2:**
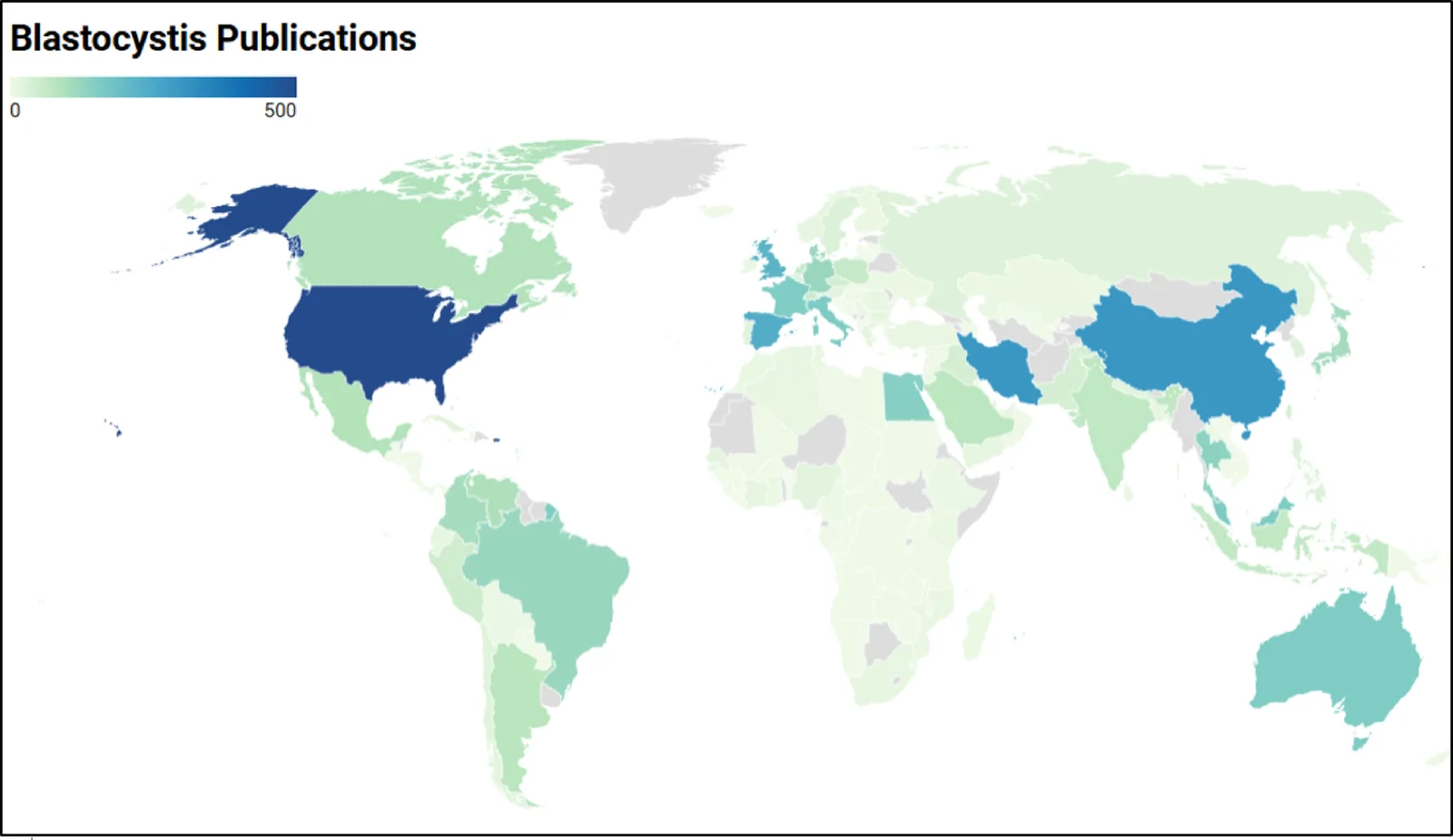
Geographic distribution of scientific publications on *Blastocystis* spp. The map represents the total number of indexed articles per country according to the Scopus database (up to 2025). Color intensity indicates publication volume

The geographic distribution of scientific publications on *Blastocystis* spp. reveals an uneven global pattern. As shown in Fig. 2, countries such as the United States, Iran and China lead in publication volume, followed by several European and Southeast Asian nations. This concentration reflects both research capacity and the local epidemiological burden of the parasite. In contrast, large regions of Africa and Latin America remain underrepresented, which may be associated with limitations in scientific infrastructure, as well as reduced research funding or lower visibility in international databases.

**Fig. 3:**
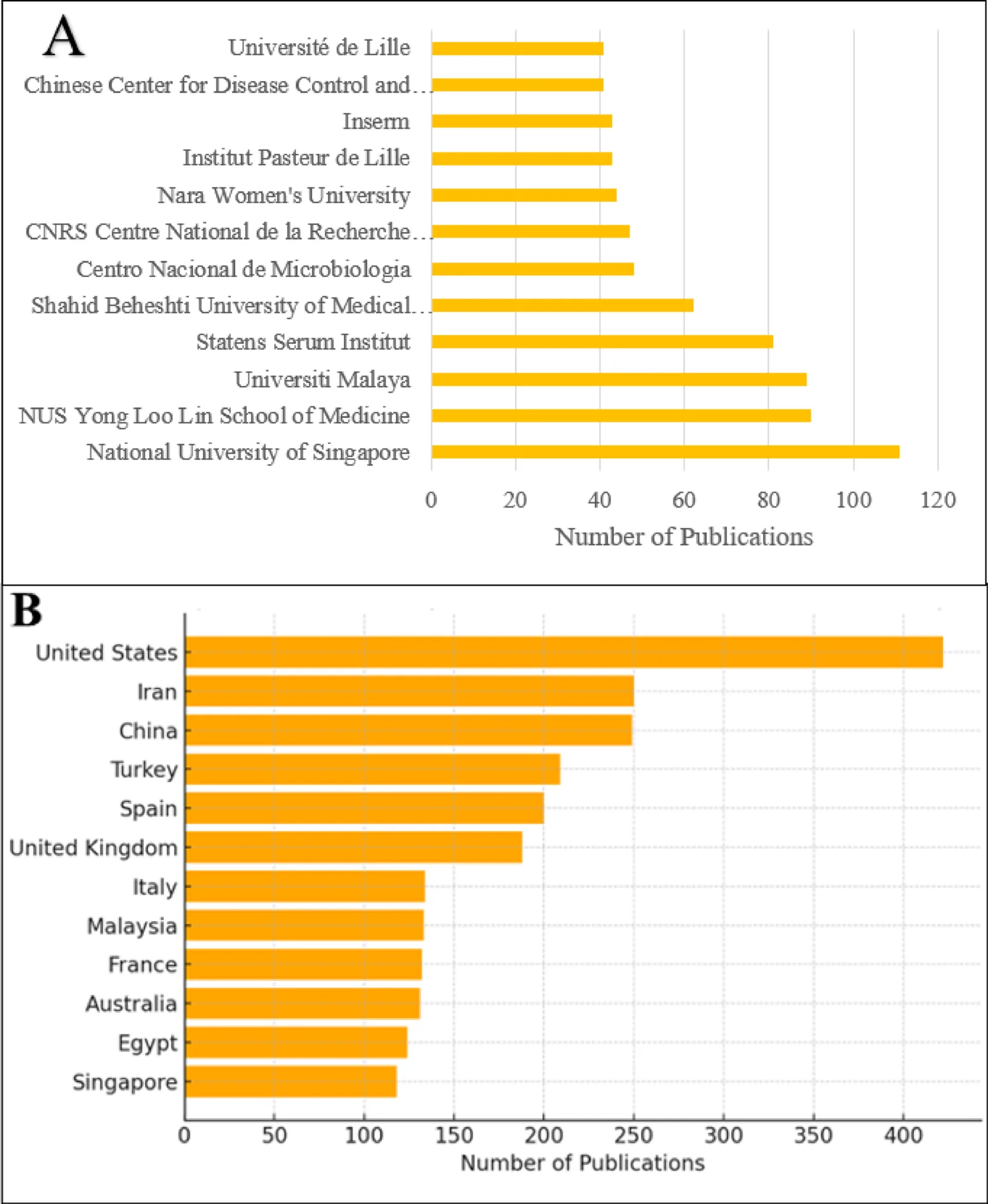
Scientific output on *Blastocystis* spp. by institution (**A**) and by country (**B**). **A** The twelve institutions with the highest number of publications. **B** The twelve countries with the highest cumulative output, based on Scopus data (up to 2025).

Consistent with the patterns observed in Figs. 2 and 3B shows the number of articles produced by the countries with the highest scientific output on *Blastocystis spp*. The United States clearly stands out, with a total of 422 publications, consolidating its position as the leading international reference in this field. It is followed by Iran (250), China (249), Turkey (209) and Spain (200), all of which show substantial contributions to *Blastocystis spp* research. The United Kingdom, Italy, Malaysia and France form a second group with slightly lower but still relevant publication counts. The top twelve is completed by Australia, Egypt and Singapore, which have also contributed consistently to advances in this area. All of these countries are included in Table 2, which incorporates demographic and economic indicators such as population size and GDP per capita to contextualize research intensity relative to national capacity. Population size and GDP per capita data used to contextualize national research output were obtained from the World Bank Open Data repository.

When considering the number of publications per million inhabitants (P/N), countries with relatively small populations such as Singapore, Spain, Malaysia and Denmark exhibit particularly high research intensity in *Blastocystis* spp. studies. Singapore exceeds 20 publications per million inhabitants, while Denmark, Malaysia and Spain all exceed four, reflecting a high degree of thematic specialization (Table 2).

**Table 2:** Number of scientific publications on *Blastocystis* spp. by country, together with total population (millions, late 2025), GDP per capita (USD) in 2025 and publications per million inhabitants (P/N)

Country	Publications	Population (Millions)	*P*/*N* (Publications per million)	GDP per capita (USD)
United States	422	334.0	1.26	80,300
Iran	250	90.6	2.76	5668
China	249	1420.0	0.18	12,700
Turkey	209	86.1	2.43	15,463
Spain	200	47.9	4.18	28,830
United Kingdom	188	67.0	2.81	47,200
Italy	134	58.9	2.28	34,373
Malaysia	133	33.0	4.03	11,700
France	132	65.0	2.03	43,700
Australia	131	26.0	5.04	64,600
Egypt	124	111.0	1.12	4300
Singapore	118	5.9	20.0	82,700

These indicators allow comparison of relative research intensity across countries, adjusted for population size and economic capacity.GDP, Gross Domestic Product; USD, United States Dollars; P/N, Publications per million inhabitants.

A more detailed institutional-level analysis reveals that a large proportion of *Blastocystis* spp. research output is concentrated in a relatively small number of highly specialized research centres (Fig. 3A). Some of these institutions coincide with the affiliations of the most productive authors, such as the National University of Singapore, the Statens Serum Institut and the CDC in the United States. Others appear prominently in the institutional ranking without being directly associated with the most prolific individual authors, suggesting a broader diversification of active research teams within these institutions.

When this indicator is analysed in conjunction with GDP per capita, distinct profiles of scientific production emerge. On one hand, high-income countries such as the United States, France and Australia combine strong economic capacity with substantial research output, although their relative intensity (P/N) is not always proportional to available resources. On the other hand, middle-income countries such as Iran, Egypt and Malaysia achieve notable levels of adjusted productivity despite more limited economic capacity. This contrast suggests that *Blastocystis* spp. research in some contexts may be driven more by local epidemiological needs than by resource availability.

**Fig. 4:**
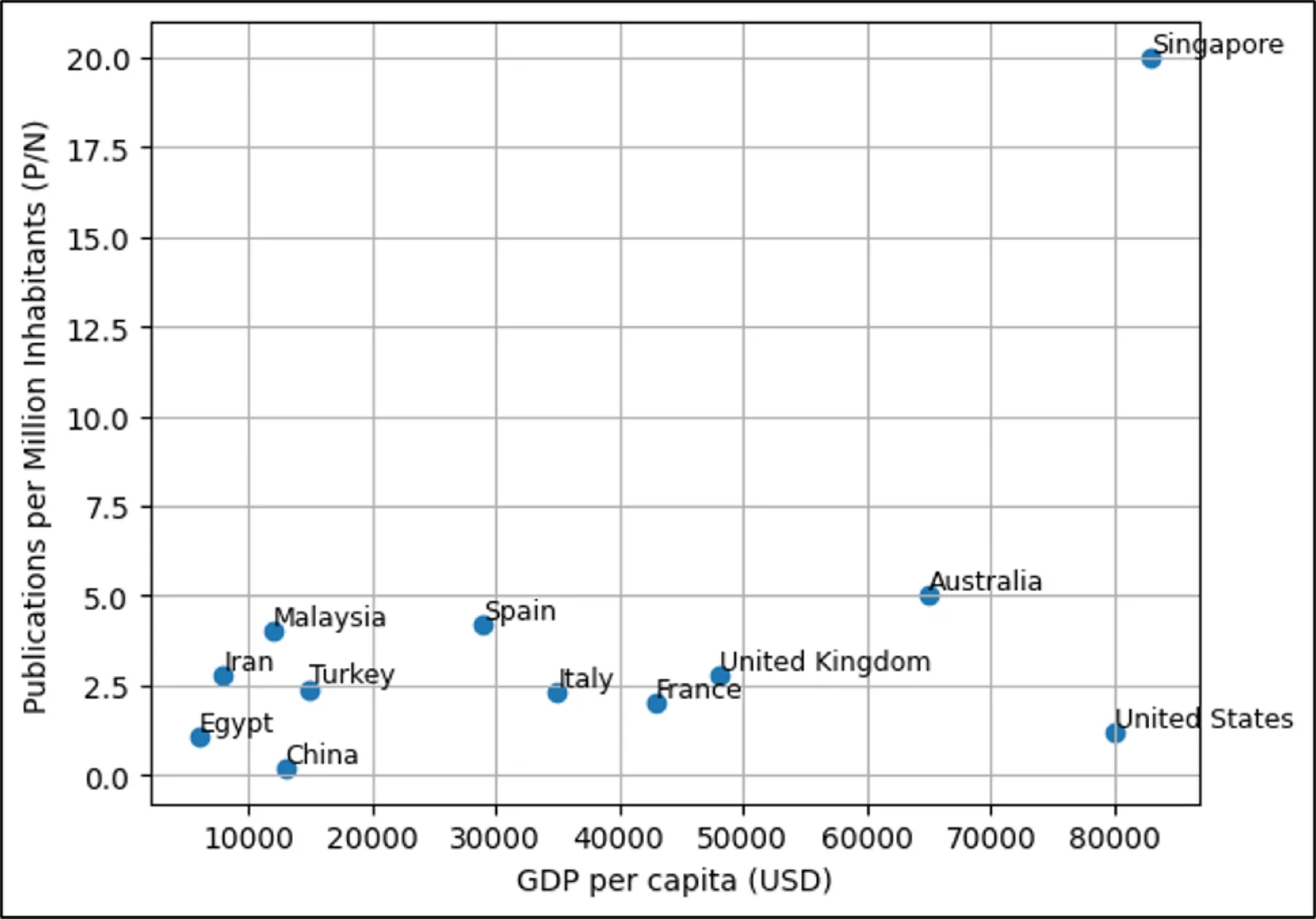
Relationship between GDP per capita and the number of publications per million inhabitants (P/N) on *Blastocystis spp*. The graph highlights countries with high relative research intensity adjusted for national wealth. Although high-GDP countries tend to publish more, several middle-income countries also show remarkable scientific productivity relative to population size

These patterns are summarized in Fig. 4, where the relationship between P/N and GDP per capita allows the identification of different national profiles, ranging from countries with high economic capacity and sustained output to those with elevated research intensity relative to population size, possibly reflecting specific public health priorities.

### Keyword Analysis: Molecular Approaches and Coinfections

**Fig. 5:**
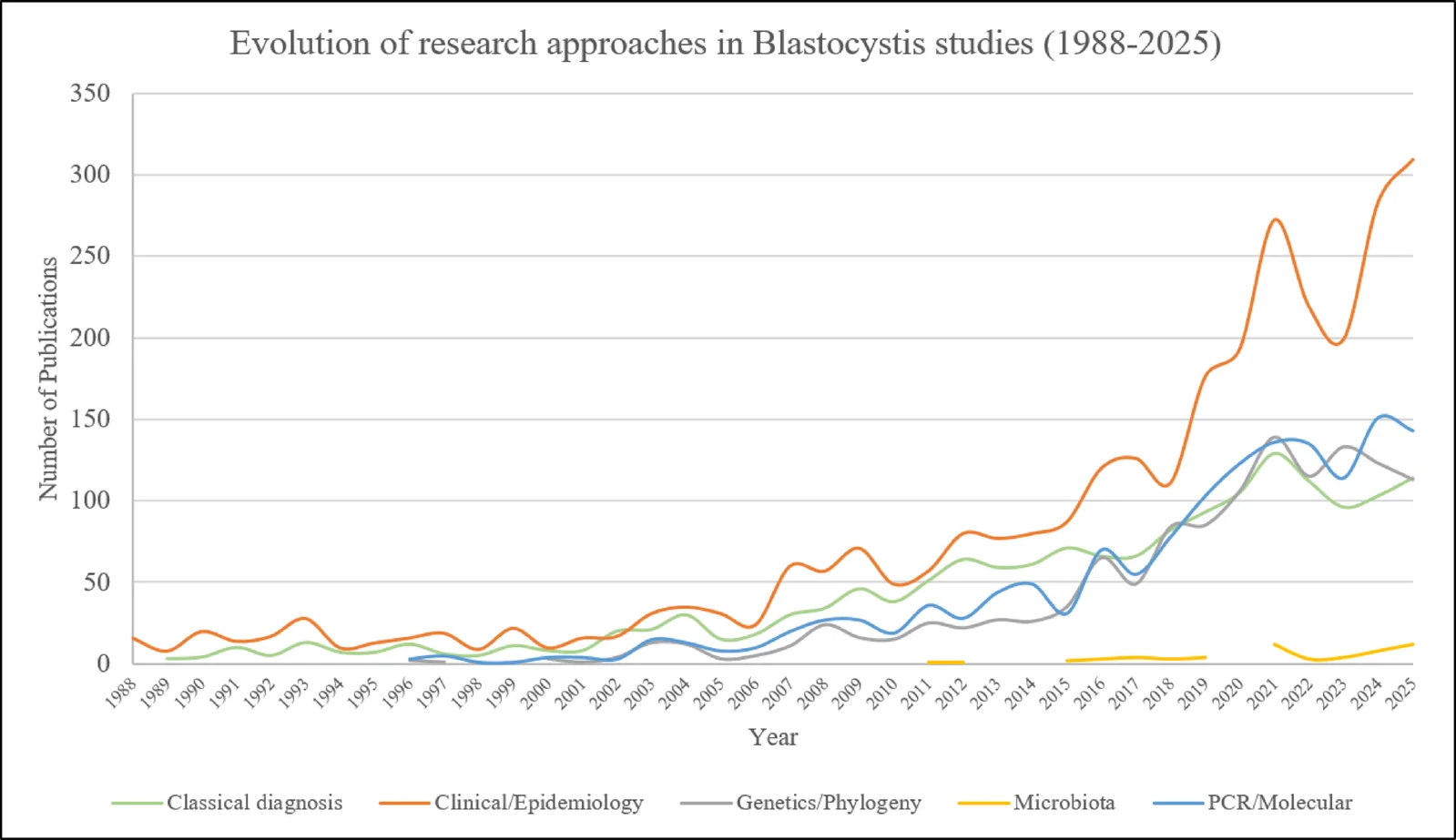
Temporal evolution of research approaches in *Blastocystis* spp. studies (1988–2025). The figure shows the annual number of publications classified into five main categories: classical diagnosis, clinical and epidemiological studies, PCR(Polymerase Chain Reaction)/molecular approaches, genetics/phylogeny and microbiota-related studies. Early research was dominated by classical diagnostic methods, while the introduction of molecular techniques from the late 1980s onwards was associated with a sustained increase in PCR-based and genetic studies. From the 2000s, clinical and epidemiological research expanded markedly, and in recent years a research line focused on the intestinal microbiota has emerged, reflecting a shift towards more integrative and ecological approaches to the study of *Blastocystis* spp.

To characterise the evolution of research strategies in *Blastocystis* spp. studies, keywords were classified into five main methodological categories and analysed over time. This framework provides a structured overview of how scientific focus has shifted across diagnostic, molecular, clinical and ecological domains. The annual distribution of publications within these categories is presented in Fig. 5.

During the earliest decades of the analysed period (from 1988), scientific output was limited and almost exclusively dominated by classical diagnostic studies, primarily based on microscopy and conventional parasitological methods. This stage is characterized by low methodological diversification and a small number of annual publications.

From the late 1980s onwards, a progressive increase in the number of studies is observed, coinciding with the emergence of molecular approaches, initially reflected in the growth of PCR/molecular studies. This period marks the beginning of a methodological transition, in which DNA amplification and analysis techniques began to complement classical diagnostic approaches.

Throughout the 1990s and the early years of the twenty-first century, a sustained increase in PCR/molecular and genetics/phylogeny categories is evident, accompanied by a parallel rise in clinical and epidemiological studies. This pattern suggests a progressive integration of molecular information into investigations of prevalence, genetic diversity and the potential clinical relevance of *Blastocystis* spp.

From approximately 2011 onwards, scientific production shows a marked increase, with a clear predominance of clinical/epidemiological studies, followed by PCR/molecular and genetics/phylogeny approaches, which reach high and relatively stable values. During this period, the microbiota category clearly emerges; although its absolute volume is lower than that of other categories, it exhibits a growing trend in recent years.

**Fig. 6:**
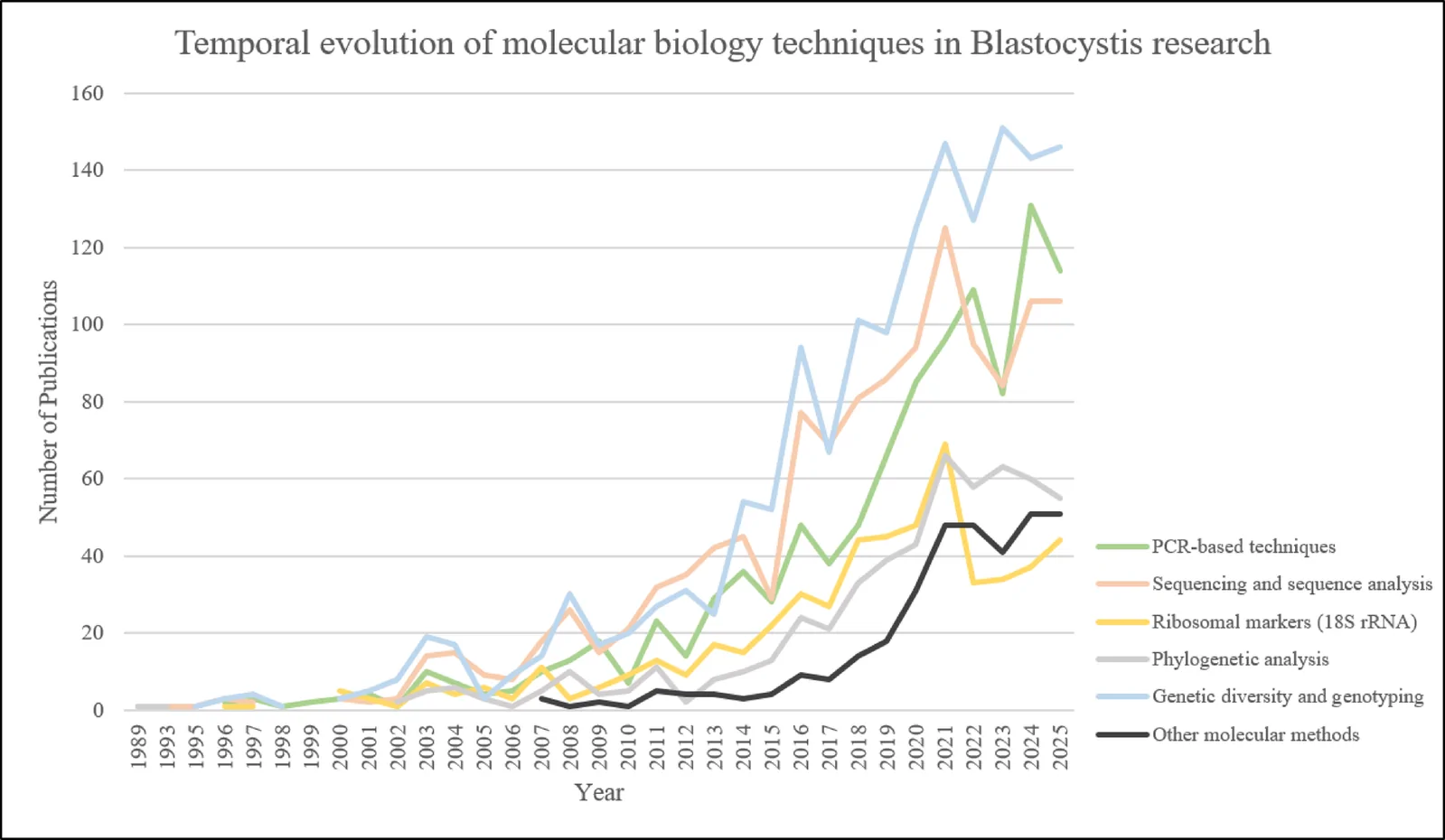
Trends in the use of molecular terms in publications on *Blastocystis* spp. (1988–2025). The graph shows the annual number of indexed articles including terms related to genetic and molecular biology techniques, such as “PCR”, “sequencing”, “genetic variability” or “phylogenetic analysis”. A sustained increase is observed from the late 1990s, with a recent peak followed by stabilization, reflecting the rise of genomic approaches applied to the study of the parasite. SSU rRNA: Small Subunit Ribosomal RNA

Building upon the general trends identified in Fig. 5, a more detailed examination of molecular terminology was conducted in order to explore the internal dynamics of methodological change within the field. The combined analysis of keyword frequency and temporal trends related to molecular biology techniques in *Blastocystis* spp. studies reveals a progressive shift in the methodological approaches employed over time (Fig. 6). Among the analysed categories, the characterization of genetic diversity and subtype genotyping represents the most prominent thematic focus across the analysed years, highlighting the sustained interest in describing the intraspecific variability and population structure of *Blastocystis* spp.

From the late 1980s onwards, a gradual incorporation of molecular biology and genetics-related terms can be observed, coinciding with the introduction of PCR-based techniques and the use of ribosomal markers for the initial identification and typing of the parasite. These approaches dominate the early stages of the analysed period, reflecting the central role of PCR as a fundamental tool for the detection, amplification and genotyping of *Blastocystis* spp.

From the 2000s, and particularly from 2011 onwards—coinciding with the first sequencing of a complete *Blastocystis* spp. subtype genome [36]—there is a marked acceleration in the use of terms associated with sequencing and sequence analysis, such as *sequencing*, *sequence analysis*, *phylogeny*, *ribosomal RNA*, *SSU rRNA* and *subtype *[37-39]. Although PCR-based techniques remain the most frequent in terms of cumulative keyword counts, the temporal analysis shows that sequencing-based approaches exhibit a more pronounced increase in recent years, eventually surpassing PCR in the most recent stages.

This divergence between the historical weight of PCR and the recent predominance of sequencing reflects a methodological transition within the field. PCR-based methods have served for decades as the reference technique for the initial identification and genotyping of *Blastocystis* spp., whereas the expansion of sequencing technologies has facilitated their application in studies focused on genetic diversity and phylogenetic relationships among subtypes. Overall, these results demonstrate the consolidation of molecular biology as a central axis of *Blastocystis* spp. research, with an increasing emphasis on detailed genetic characterization of the parasite.

**Fig. 7:**
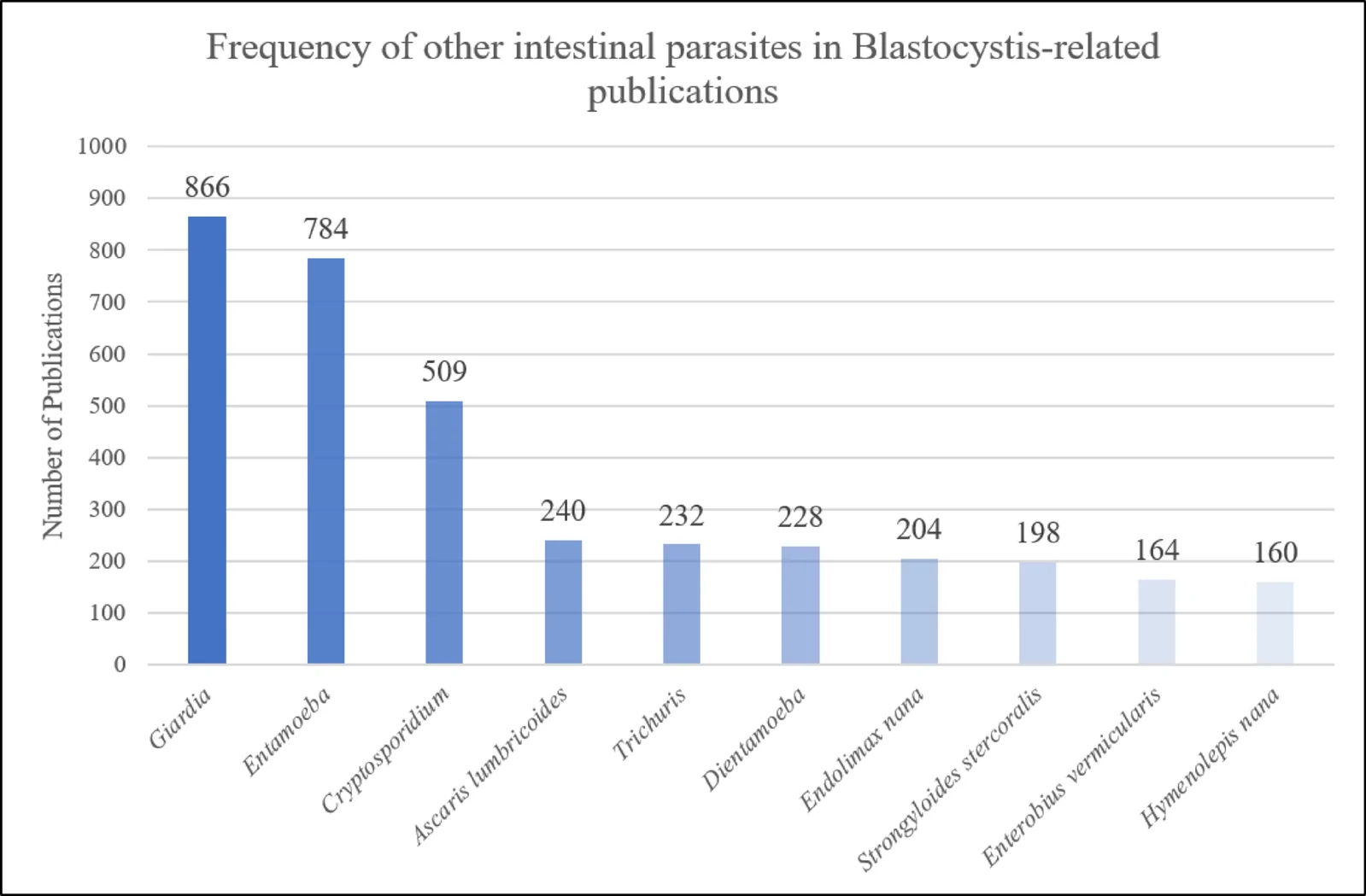
Frequency of other intestinal parasites reported in scientific publications on *Blastocystis* spp. The bar chart represents the absolute number of indexed articles mentioning, in addition to *Blastocystis* spp., other intestinal protozoa and helminths. *Giardia* and *Entamoeba* are the most frequently reported taxa, followed by *Cryptosporidium*, indicating a recurrent pattern of coinfection in epidemiological and clinical studies

In addition, co-occurrence analysis of keywords shows that *Blastocystis* spp. is rarely investigated in isolation. Its frequent co-occurrence with other intestinal parasites indicates that many studies examine *Blastocystis* spp. alongside globally prevalent protozoa such as *Cryptosporidium parvum* and *Giardia intestinalis*, as well as helminths and protozoa commonly found in low-income or resource-limited settings, such as *Ascaris lumbricoides* or *Entamoeba histolytica* (Fig. 7). This simultaneous presence suggests that many investigations are conducted in epidemiological contexts characterized by high parasite burdens, where co-parasitism is common, particularly among vulnerable populations with poor hygienic and sanitary conditions. The frequent use of terms such as *polyparasitism*, *intestinal parasites* or *coproparasitological study* further supports this interpretation.

Overall, the keywords used in the literature on *Blastocystis* spp. reflect both a strong interest in the molecular characterization of the parasite and its study within the broader framework of multiple intestinal infections, underscoring its relevance in public health and comparative parasitology contexts.

This distribution highlights the robust thematic clusters obtained, which perfectly align with the standard methodological frameworks for structural and conceptual evolution in science mapping [40, 41].

## Discussion

The present bibliometric study demonstrates a sustained and accelerated growth in scientific production on *Blastocystis* spp. over the last century, with a particularly marked increase from the early twenty-first century onwards and, more notably, since 2015. This quantitative trend is accompanied by a profound qualitative transformation in the conceptual and methodological approaches employed, as clearly evidenced by the chronological trajectory of research strategies (Fig. 5).

During the earliest decades analysed, research on *Blastocystis* spp. was constrained by the technical limitations inherent to classical parasitology, which explains the almost exclusive predominance of morphological diagnosis and descriptive studies. In this context, the parasite was interpreted heterogeneously, oscillating between being considered an intestinal commensal and an opportunistic pathogen, in the absence of adequate tools to address its biological diversity or clinical variability.

The introduction and progressive widespread adoption of PCR and molecular analysis techniques from the late 1980s onwards represented a decisive turning point in the field. This initial upsurge in molecular studies chronologically preceded and facilitated the subsequent development of research focused on genetics and phylogeny (Fig. 5). Amplification and sequencing of the SSU rRNA gene overcame the limitations of conventional microscopy, revealing a previously underestimated prevalence and genetic diversity that led to the definition of multiple distinct subtypes [42, 43, 44]. Consequently, *Blastocystis* spp. ceased to be regarded as a homogeneous entity and came to be understood as a complex of lineages with potentially distinct biological and clinical implications. This technological transition was structurally reinforced by the consolidation of international centers of excellence—such as Stensvold (Denmark), Tan (Singapore), Carmena (Spain) and Viscogliosi (France)—playing a central role in the transition from descriptive approaches to molecular, phylogenetic and functional studies [43, 45, 46].

From a geographic perspective, scientific production is concentrated in a limited number of countries with high research capacity, such as the United States, China, Iran and several European nations, in line with patterns observed in other areas of parasitology [41]. However, population-adjusted analyses reveal that smaller countries, such as Singapore, Denmark and Spain, exhibit particularly high research intensity, suggesting that productivity in this field depends not only on macroeconomic factors but also on the presence of highly specialized research groups and well-established research lines.

More recently, bibliometric data reveals the rapid emergence of an additional conceptual shift: the study of *Blastocystis* spp. within the context of the human gut microbiome (Fig. 5). This integration has fundamentally reframed the classical, binary pathogen-vs-commensal debate. Accumulating metagenomic and observational evidence consistently links *Blastocystis* spp. colonization to higher bacterial diversity and microbial profiles compatible with eubiosis, particularly in asymptomatic individuals [24].

Rather than being considered an intrinsic and uniform property of the parasite, pathogenicity is now increasingly understood as a context-dependent phenomenon heavily modulated by the host’s baseline immunity and microbial ecosystem composition [44].

This ecological perspective is strongly supported by recent experimental models providing direct evidence of a bidirectional cross-talk between *Blastocystis* spp. and surrounding bacteria. For instance, Deng & Tan [46] demonstrated, using in vitro models, that subtype ST4 can modulate the growth and composition of bacterial communities, while Rajamanikam et al. [21] showed that the accompanying microbiota can modify phenotypic and potentially pathogenic traits of *Blastocystis* ST3, and that the presence of the parasite, in turn, alters the composition and metabolic functions of the gut microbiota. These results provide a robust explanatory framework for the heterogeneity observed in clinical and epidemiological studies, acknowledging the difficulty of proving causality in this context.

Taken together, our bibliometric analysis highlights a profound qualitative transformation, tracing how *Blastocystis* spp. research has evolved from a narrow, niche topic within classical parasitology into a dynamic, multidisciplinary field. The chronological trends (Fig. 5) demonstrate a clear paradigm shift: studies have progressively moved away from isolated morphological and conventional descriptive frameworks to integrate molecular, genetic, and ecological perspectives. This transition positions *Blastocystis* spp. not only as a pathogen of clinical interest but also as an emerging biological model for studying the intricate, context-dependent evolutionary dynamics within the human intestinal ecosystem.

Looking forward, the increasing incorporation of high-throughput microbiome perspectives suggests that the frontier of *Blastocystis* spp. research will transcend basic prevalence or binary pathogenicity surveys. Future directions will increasingly rely on the integration of advanced next-generation sequencing (NGS), functional genomics, and well-defined experimental models to fully unravel the specific biological roles and clinical relevance of different subtypes. Moving toward these integrative frameworks will be critical for refining diagnostic accuracy, predicting clinical outcomes based on host-microbiota profiles, and ultimately translating these ecological insights into tailored therapeutic and preventive strategies within the realm of precision medicine.

## Data Availability

All data generated or analysed during this study are included in this published article.
